# Validation of the Prospective Memory Concerns Questionnaire (PMCQ)

**DOI:** 10.3389/fnhum.2021.686850

**Published:** 2021-08-26

**Authors:** Nicole Sugden, Matt Thomas, Michael Kiernan, Michele Wilesmith

**Affiliations:** ^1^School of Psychology, Charles Sturt University, Bathurst, NSW, Australia; ^2^Marathon Health, Bathurst, NSW, Australia; ^3^Western New South Wales Local Health District, Bathurst, NSW, Australia

**Keywords:** prospective memory, questionnaire, self-report, validation, memory for intentions

## Abstract

Prospective memory (PM), the ability to remember to complete intended tasks, is essential for successfully completing activities of daily living. PM impairments are common in people with neuropathology such as acquired brain injury and dementia. These PM impairments affect individuals’ capabilities in key aspects of daily living including their health, safety, and independence. The Prospective Memory Concerns Questionnaire (PMCQ) was designed as a self-report measure to understand individuals’ concerns about their memory. This questionnaire may help identify issues with PM which in turn may assist clinicians in the targeted implementation of memory compensation strategies. The PMCQ was developed using Rasch and classical test methodologies, with subscales measuring frequency of forgetting behaviors, memory concerns, and retrieval failures. The current study aimed to confirm the factor structure of the PMCQ for use in adults in the general population. The study also aimed to examine relationships between the PMCQ and naturalistic performance-based measures of PM to determine how the self-report PMCQ could be used in conjunction with performance-based measures. A community dwelling sample of 558 adults completed the PMCQ, an event-based naturalistic PM task, and time-based naturalistic PM task. Confirmatory factor analyses (CFAs) indicated that a higher order model with three subscales containing 35 items produced acceptable fit [RMSEA = 0.056 (90% CI 0.054, 0.060), SRMR = 0.062, CFI = 0.915, TLI = 0.909] The PMCQ demonstrated good internal consistency (total α = 0.95, subscales: α = 0.88–0.89). The Forgetting Behaviors subscale significantly correlated with performance on the event-based naturalistic PM task (*r* = −0.14, *p* < 0.01). The Memory Concerns and Retrieval Failures subscales did not correlate significantly with performance-based PM tasks. These findings suggest that the PMCQ may be best suited for assessing individuals’ concerns about their forgetting behaviors and identifying appropriate compensation strategies or support services. It is recommended that the PMCQ be used alongside performance-based PM tasks and other cognitive measures to comprehensively assess PM. It was concluded that the PMCQ is a suitable measure for use in adults in the general population. Further validation research of the PMCQ in general population and clinical samples will determine the measures’ sensitivity and specificity in identifying PM impairments.

## Introduction

Prospective memory (PM) involves remembering to fulfill an intention at a future point in time (McDaniel and Einstein, 2007). On any given day, we are engaged in multiple PM tasks as we complete household chores, carry out work-related tasks, meet social obligations, and maintain our health. PM failures have been found to be reported more frequently than other cognitive errors (Haas et al., 2020). Moreover, PM failures have been linked to poor outcomes in relation to medication adherence (Zogg et al., 2012) and independent functioning (Hering et al., 2018). This has particularly been so for those with cognitive impairments resulting from acquired brain injury (Hogan et al., 2016), traumatic brain injury (Shum et al., 2002, 2011), or neurodegenerative conditions such as dementia (Van Den Berg et al., 2012).

The effects of PM errors on daily functioning may be reduced through restorative cognitive training or the adoption of memory compensation strategies such as note-taking or assistive technology (for a review of interventions in brain injury, see Raskin et al., 2018). For example, Evans et al. (2003) found that memory aid usage predicted independence in individuals with brain injury. Several restorative (Raskin and Moore Solberg, 2009; Raskin et al., 2019) and compensatory interventions (Radford et al., 2012; Evald, 2017; Withiel et al., 2019) have also shown promising improvements in PM in individuals with brain injury, although more research is required to establish their generalized and long-term effectiveness. Implementing appropriate interventions requires a comprehensive assessment of PM performance and other related cognitive functions in order to determine the type and frequency of lapses that need to be targeted (Fish et al., 2010).

Assessment of PM behaviors, failures, and intervention outcomes is difficult, as each PM task is unique. PM tasks vary considerably in the duration of retention intervals (Hicks et al., 2000), number and nature of ongoing tasks (Smith et al., 2012), characteristics of retrieval cues and context (Ihle et al., 2013), task importance (Altgassen et al., 2010), influence of interruptions (Kliegel et al., 2008), and whether strategies are used to assist retrieval (Chen et al., 2015), amongst many other factors. Similarly, PM task characteristics will determine which cognitive resources are required to fulfill the intention. For example, retrospective memory (RM) processes may be used to encode and retrieve details of about how, where, and when the intention needs to be carried out; working memory may be involved in retaining multiple intentions at once; and executive functioning processes may be deployed to monitor the environment for retrieval cues or for switching between ongoing and PM tasks (Clune-Ryberg et al., 2011; Schnitzspahn et al., 2013). Therefore, the challenge in assessing PM behaviors and intervention outcomes is in developing measures that can capture this multidimensionality of everyday PM tasks, whilst being able to identify specific task, context, cognitive, and metacognitive factors that may be contributing to PM failures.

Various performance-based PM tasks have been developed for the purposes of identifying PM impairments and evaluating intervention outcomes. Computerized laboratory-based tasks require participants to respond to PM target words that are embedded in ongoing cognitive tasks (Einstein and McDaniel, 1990). The advantage of these tasks is that individual variables such as cue salience (Smith et al., 2007) or ongoing task load (Marsh et al., 2002) can be controlled for in order to investigate factors underlying PM performance. Although laboratory-based tasks can isolate independent variables that contribute to PM failures, they lack ecological validity (Philips et al., 2008). Like laboratory-based tasks, naturalistic tasks permit the control of certain variables, but they can be carried out in everyday settings (e.g., remembering to make a telephone call; Kvavilashvili and Fisher, 2007). Naturalistic tasks can also be incorporated into a neuropsychological assessment in clinical settings (e.g., the hidden belonging subtest of the Rivermead Behavioral Memory Test requires individuals to remember to ask for a hidden belonging to be returned at the end of the assessment; Wilson et al., 1985). Though, when embedding PM tasks within other neuropsychological tasks caution is needed to ensure that task demands do not draw cognitive resources away from other tasks (Marsh et al., 2002). As naturalistic tasks typically include only a single trial and use binary scoring (i.e., correct or incorrect) that provides little information about reasons for forgetting, they provide limited information to inform intervention selection or evaluate outcomes (Uttl and Kibreab, 2011).

To overcome these limitations, several standardized PM tests have been developed. These include the Cambridge Prospective Memory Test (CAMPROMPT; Wilson et al., 2005), Memory for Intentions Screening Test (MIST; Raskin and Buckheit, 1998), and Royal Prince Alfred Prospective Memory Test (RPA-ProMem; Radford et al., 2011). These PM tests include a set of naturalistic tasks that assess different components of PM (e.g., time versus event-based tasks or short versus long retention intervals). Scoring methods allow administrators to distinguish between distinct types of errors on PM tasks and account for memory aid usage. Virtual Week, a board game-style test where participants complete imaginary tasks as they progress along a game board that represents virtual days and times of the week, has also been used in research and clinical settings to assess PM (Rendell and Henry, 2009). The advantages of these tests are that their procedures are standardized, alternate forms can be used for repeated testing, they are psychometrically reliable and valid, and normative data is available. However, on their own, these performance-based measures cannot capture the complexity of everyday PM due to the artificial settings in which they are used, the limited range of variables tested, and their ability to only measure observable PM behaviors.

To provide a more comprehensive assessment of PM, performance-based measures of PM can be complemented with self-report measures of PM. Self-report measures are often criticized for being subjective and limited by self-report biases and the influence of factors such as personality (Hertzog and Pearman, 2014). However, they provide important information from the individual’s perspective that can be used to guide treatment planning (Roche et al., 2007). Self-report measures of PM can assess forgetting on unobservable PM tasks (e.g., remembering to tell a friend about a TV show they watched). They can also monitor forgetting behaviors that are of the greatest concern to the individual, internal strategies used to facilitate remembering, and reasons for forgetting such as lack of motivation or distractedness (Shum et al., 2002; Chau et al., 2007). Informant-report versions of these questionnaires that are completed by a proxy can also help identify when individuals lack awareness of their memory problems (Roche et al., 2002; Crawford et al., 2006). Thus, as self-report PM questionnaires ask individuals to reflect on their actual previous performance on a variety of everyday PM tasks, they capture some of the complexity of real-world PM that performance-based measures cannot (Chau et al., 2007).

There are currently four self-report PM scales available:

• The 52-item Prospective Memory Questionnaire (Hannon et al., 1995) measures forgetting on long-term episodic, short-term habitual, internally cued tasks as well as techniques to remember.
• The 16-item Prospective and Retrospective Memory Questionnaire (PRMQ; Smith et al., 2000) measures the frequency of common PM and RM failures.
• The Comprehensive Assessment of Prospective Memory (CAPM; Shum and Fleming, 2014) measures both the frequency and importance of forgetting on 39 PM tasks, and includes an additional 15 items measuring cognitive processes involved in remembering.
• The 16 item Brief Assessment of Prospective Memory (BAPM; Man et al., 2011) adapted the CAPM as the full assessment may be too fatiguing for individuals with brain injury. This subset of 16 items measures basic and instrumental activities of daily living that are relevant to individuals with brain injury.

Each of these PM questionnaires captures different aspects of PM, which means that each has gaps in what it can measure. Moreover, each scale has been utilized for different purposes (see Sugden et al., 2021 for a review). For example, the PRMQ is frequently used in research settings due to its brevity and ability to differentiate between PM and RM failures. The PMQ, with its techniques to remember scale, has been used to measure everyday forgetting, particularly in drug and alcohol populations. Whereas the CAPM and BAPM have been primarily used in other clinical populations (e.g., brain injury, mild cognitive impairment, and ADHD). As each of these scales has its own niche area, there is room for the development of alternative PM self-report measures that may target aspects of PM (e.g., metacognitive concerns and use of retrieval cues) that have not been captured in these existing measures (McDaniel and Einstein, 2007). This would allow clinicians to have greater choice when looking for self-report PM measures to complement performance-based measures and more comprehensively assess PM.

The Prospective Memory Concerns Questionnaire (PMCQ; Sugden, 2015) was developed to address the aforementioned gaps in the measurement of self-reported PM. This questionnaire was designed to capture a broad range of dimensions involved in everyday PM, with a focus on individuals’ concerns about their PM. Through a review of the PM literature, a pool of 340 items was created to extensively capture dimensions of PM. Items underwent an expert review using a panel of individuals (*n* = 36) who had frequent contact with individuals with PM impairments (e.g., carers of individuals with dementia, aged care-nurses, psychologists, and specialists in aged care assessment teams). The expert panel rated items on their relevance to the measurement of PM as well as the items’ readability. Item relevance ratings guided item removal, although these ratings were cross-referenced with item content to ensure that each PM dimension was represented in subsequent item analyses. Item readability ratings were then used to refine the 135 items retained in the item pool. These remaining items underwent Principal Component Analysis and Rasch analyses to refine items and subscales that maximized reliability, content validity, assessment of a variety of PM dimensions, individual item functioning, and that targeted a wide distribution of person ability with items of differing difficulty levels. These analyses resulted in a reliable 42-item scale (α = 0.90) with three subscales: Forgetting Behaviors (16 items, α = 0.77), Memory Concerns (14 items, α = 0.85), and Retrieval Cues (12 items, α = 0.74). Validation of the scale found that a CFA replicated the three-factor solution and that the PMCQ had convergent validity with the PM scale of the PRMQ (*r* = 0.80) and divergent validity with the RM scale (*r* = 0.69). When PMCQ scores of a general population sample (*n* = 207) were compared to a reference group comprised of individuals with brain injury, dementia, and mild cognitive impairment (*n* = 29), the reference group reported significantly more concerns on the PMCQ, Forgetting Behaviors, and Memory Concerns subscales, suggesting that the PMCQ had predictive validity and was able to differentiate memory concerns of those with cognitive impairments from those in the general population.

The aim of the current study was to further validate the PMCQ using a large sample, to confirm its factor structure for use with adults in the general population. As it is intended for the PMCQ to be used within a comprehensive assessment of PM in conjunction with brief performance-based measures, we evaluated relationships between the PMCQ and an event-based naturalistic PM task and time-based naturalistic PM task to determine how the PMCQ could be used alongside these tasks. Our recent review of self-report PM measures established that self-report PM scales only have weak correlational relationships with performance-based measures and are likely to measure different PM constructs (Sugden et al., 2021). We therefore predicted only weak relationships between self-report and performance-based measures in this study, whereby each measure serves a unique purpose in the measurement of PM.

## Materials and Methods

### Design

This longitudinal study was carried out in two parts. In part one, participants completed an online questionnaire with an embedded event-based naturalistic PM task. Part two was an optional time-based naturalistic PM task.

### Participants

A total of 635 participants were recruited for this study. Of these, 456 participants were recruited through social media and free participant recruitment websites. An additional 84 participants were recruited through the Charles Sturt University first year psychology student research participation pool. To reduce a gender skew in the dataset, Prolific Academic was also used to recruit 95 male participants who were paid the equivalent of £1.25 for their time. Data from four participants was excluded due to participants not meeting the minimum age criteria of 17 years. An additional seven responses were deleted due to invalid data (i.e., speeded responses) and 66 cases were removed due to participants withdrawing from the study prior to completion of the questionnaire (43 of these cases did not proceed past the demographic questions).

Table 1 presents the demographic data for the full sample as well as the sample for part 2 of the study. As can be seen in the table, part 2 participants were significantly older than the overall sample. However, the samples did not differ in terms of gender or educational levels.

**TABLE 1:** Demographic data for full sample and part 2 subsample.

	Full sample	Part 2 sample	*p*
*N (%)*	558 (100)	219 (39)	
Age (*M, SD*)	37.28 (16.29)	41.96 (16.72)	<0.001
Age range	17–83	17–78	
Gender (*n, %)*			0.128
Male	232 (41.6)	99 (45.2)	
Female	320 (57.3)	119 (54.3)	
Gender fluid	2 (0.003)	0 (0.0)	
Other	3 (0.005)	0 (0.0)	
Preferred not to specify	1 (0.001)	1 (0.5)	
Education (*%*)			0.362
Did not complete high school	3.4	2.7	
Completed high school	21.3	17.4	
Vocational qualification	8.6	8.7	
Diploma	14.2	14.2	
Bachelor’s degree	34.8	36.1	
Postgraduate qualifications	17.7	21.0	

Participants’ primary language was English, with approximately 85% of participants residing in Australia. The remaining participants were from the United States, United Kingdom, and Europe.

### Materials

The online questionnaire in part one of the study was administered online via the Qualtrics platform. This questionnaire included demographics questions, the PMCQ, and an embedded event-based naturalistic PM task. The questionnaire also included the Australian Personality Inventory (Murray et al., 2009) and Arousal Predisposition Scale (Coren and Mah, 1993) as a part of a larger study, however, the findings of these scales are not reported here.

#### PMCQ

Self-reported concerns about PM were measured using the PMCQ (Sugden, 2015). The scale included 42 items measured on a rating scale of 0 (*never*) to 3 (*always*), with higher scores indicating greater concerns about PM.

#### Event-Based Naturalistic PM Task

An event-based naturalistic PM task was embedded into the questionnaire. Participants were given the following instructions for the event-based task, highlighted in bold text: “Once you have completed all of the questions, you will see a comment box. In that comment box, please type in whether you are left or right-handed.” This comment box was presented at the end of the questionnaire, with the instruction to “please provide any comments here.” Successful event-based PM performance where participants typed their handedness into the comment box was scored as one. An incorrect score of zero was given when the comment box was left blank, or the comment did not indicate handedness.

#### Time-Based Naturalistic PM Task

Part two of the study was a time-based naturalistic PM task that required participants to remember to send a text message. The time-based task was optional to ensure that only participants who consented, and therefore intended to complete the task, were included. Participants who consented were instructed to text the word “hello” to the researcher’s phone between midday and midnight the following day. Participants were then given the researcher’s phone number and told to record it in any way they wished in order for them to be able to send the message the following day. Participants were also asked to provide the phone number that they would send the text message from to allow matching of questionnaire data to responses on the time-based task. Participants who remembered to text the word “hello” within the 12-h window the following day received a score of two. Those who remembered to send a text message, but did not text the word “hello,” sent the message at the incorrect time, or a combination of both, received a score of one. A score of 0 was assigned to participants from whom a message was not received.

### Procedures

Approval for the study was provided by Charles Sturt University’s Human Research Ethics Committee (protocol number H20074). Upon providing informed consent, participants completed the demographic questions. Participants then received the instructions for the event-based naturalistic PM task prior to completing the PMCQ, Australian Personality Inventory, and Arousal Predisposition Scale. The order of these three scales were counterbalanced across participants and acted as the ongoing cognitive task within which the event-based task was embedded. These 104 questionnaire items provided a retention interval of approximately 10 min for the event-based PM task. On completion of the questionnaires, participants were presented the comment box which served as the retrieval cue for the event-based PM task. Participants were then invited to participate in the second part of the study. Participants who consented to this optional task were given the instructions for the time-based naturalistic PM task (i.e., participants were asked to text the word “hello” to the researcher’s phone number between midday and midnight the following day). All participants were debriefed following participation.

### Data Analyses

IBM SPSS Statistics Version 27 (Ibm Corp., 2020) was used for data analyses. Confirmatory factor analyses (CFAs) were carried out using the Lavaan package (Rosseel, 2012) for R statistical software (R Core Team, 2020).

The four PMCQ items that were worded in a positive direction (i.e., “I remember”) were reverse scored to ensure all items were scored in a single direction (i.e., “I forget”). Item distributions on the PMCQ were assessed by calculating the mean, standard deviation, range, and z skew for each item. Internal consistency of the PMCQ and its subscales were measured using Cronbach’s alpha. Descriptive statistics for the PMCQ, event-based naturalistic PM task, and time-based naturalistic PM task were also calculated. Finally, Spearman’s rho correlations were used to examine the relationships between the PMCQ and the performance-based tasks.

A CFA was conducted to determine whether the data fit the three-factor model obtained during the development of the PMCQ. As item responses were ordinal and did not conform to a normal distribution, Diagonal Weighted Least Squares (DWLS) estimation was used. DWLS uses polychoric correlations and is argued to provide more robust estimates of model fit for ordinal, non-normal data than maximum likelihood estimation (Li, 2016). Model fit was tested using the robust chi-square (χ^2^), but as this statistic is sensitive to large sample size, additional fit measures were also included. These fit measures were the Root Mean Square Error of Approximation (RMSEA), Standardized Root Mean Squared Residual (SRMR), Comparative Fit Index (CFI), and Tucker-Lewis Index (TLI). Hu and Bentler (1999) proposed rule of thumb cut-off scores whereby RMSEA scores below 0.06, SRMR scores below 0.08, and CFI and TLI scores above 0.95 suggested good fit. However, as there is limited evidence of the suitability of these cut-off scores for ordinal data, caution should be used in interpreting models as having good fit using these fit statistics (Xia and Yang, 2019; Shi et al., 2020).

## Results

### Item Distributions

Descriptive statistics for the 42 PMCQ items were calculated (see Supplementary Table 1). For each of the 42 items, participants endorsed the full range of response categories (i.e., 0 *never* to 3 *always*). However, participants in this general population sample typically reported forgetting “sometimes” on the items, with the average response across all items being 0.91 (*SD* = 0.37). Item distributions were checked for normality, as significance tests for normality should not be applied for large samples (Ghasemi and Zahediasl, 2012). Inspection of distributions revealed that many items had distributions that were positively skewed with *z*-skew statistics ranging from 0.27 (item 11) to 21.06 (item 35; *M z skew* = 1.64, *SD* = 3.68).

### Structure of the PMCQ

The three-factor model had poor fit [χ^2^ (816) = 2817.345, *p* < 0.001, RMSEA = 0.066 (90% CI 0.064, 0.069), SRMR = 0.077, CFI = 0.851, TLI = 0.842] in the current sample. Alternative models based on the idea that a general memory concerns factor may account for PMCQ items were also tested. These included a model with all 42 items loading onto a single memory concerns factor, χ^2^ (819) = 2979.796, *p* < 0.001, RMSEA = 0.069 (90% CI 0.066, 0.071), SRMR = 0.080, CFI = 0.831, TLI = 0.830, and a model with the original three factors and a higher order memory concerns factor, [χ2 (816) = 2717.345, *p* < 0.001, RMSEA = 0.066 (90% CI 0.064, 0.069), SRMR = 0.077, CFI = 0.851, TLI = 0.842]. The original three-factor model was retained for modification as Rasch analyses in the development of the PMCQ suggested that the scale was multidimensional and that the three-factor structure provided clinical utility in distinguishing between general population and clinical participants.

### Modified PMCQ Model

The original three-factor model was retained, and modification indices were used in conjunction with an analysis of item distributions, item content, and internal consistency reliability indices to develop a modified model for the PMCQ. The Retrieval Cues subscale was renamed as Retrieval Failures to better reflect the failure-based nature of items in the revised subscale, given that high scores on this subscale indicate higher levels of retrieval failures. Based on these analyses, seven items were deleted: items 8, 26, and 36 which were reverse-scored items and measured general ability to remember to carry out tasks were deleted from the Forgetting behaviors subscale as they were highly skewed; negatively impacted reliability and model fit; and the PM dimensions that they addressed were considered to be better measured with other items in the scale. However, item 21 which was also reverse scored, was not deleted as its inclusion improved the psychometrics of the scale and the content was deemed important to the measurement of PM. Items 12, 14, 34, and 35 which related to salient reminders were also deleted from the Retrieval failures subscale. These deleted items were amongst those with the highest *z* skew statistics; they reduced reliability and model fit; and they were unlikely to discriminate between high and low memory concerns. In addition, modification indices suggested that loading four items onto different subscales would improve model fit: item 17 “*I forget to do things because I get carried away doing something else*” was moved from the Forgetting behaviors subscale to the Retrieval Failures subscale, and items 3 “*There are times when I remember that I need to do something, but I can’t remember what it is*,” 19 “*I forget to do some things that I have planned to do*,” and 20 “*I forget things that I am supposed to be doing if I am anxious or worried about something*” were moved from the Memory concerns subscale to the Retrieval failures subscale. Although modifications identified items that could be moved to improve model fit, final decisions on the loading of these items onto alternate subscales was based on the analysis of item content. The four items that were moved referred to individuals failing to retrieve the PM cue or failing to retrieve information necessary to complete the PM task. As such, the loading of these items onto the Retrieval failures scale theoretically supported the measurement of retrieval processes as outlined by Ellis (1996).

Following these modifications, the PMCQ model comprised of 35 items that loaded on to the original three factors. This model was found to have acceptable fit [χ^2^ = (557) 1560.839, *p* < 0.001, RMSEA = 0.057 (90% CI 0.054, 0.060), SRMR = 0.062, CFI = 0.915, TLI = 0.909]. However, the correlation between the Forgetting Behaviors and Retrieval Failures factors was high (*r* = 0.94) in this model. Therefore, this model was adapted to include a higher order Prospective Memory factor that accounted for the shared variance amongst these factors. This higher order model had acceptable fit [χ^2^ = (557) 1560.839, *p* < 0.001, RMSEA = 0.056 (90% CI 0.054, 0.060), SRMR = 0.062, CFI = 0.915, TLI = 0.909] and was therefore chosen as the final model.

**FIGURE 1:**
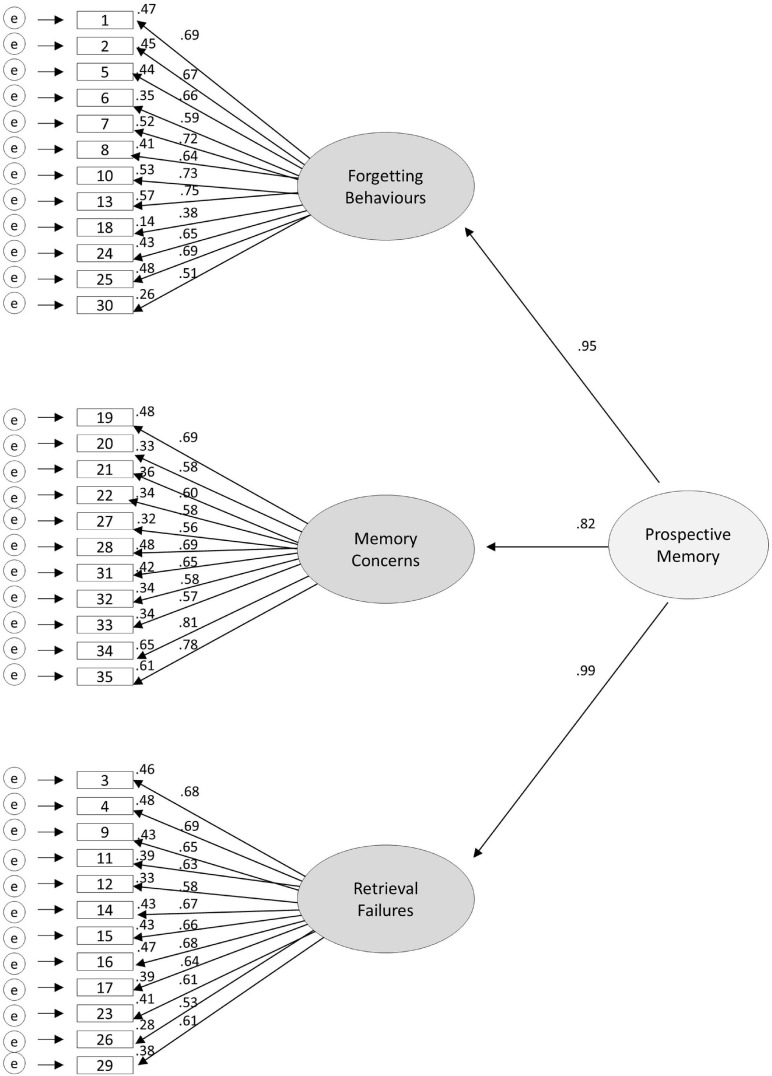
Higher order three-factor model of the Prospective Memory Concerns Questionnaire (PMCQ). Items are shown in the boxes on the left, factors are shown in the ovals, the lines illustrate the standardized loadings for factors, and the numbers at the top right of each item represents the squared multiple correlations or variance accounted for (*R*^2^) for each item.

Table 2 includes the 35 retained PMCQ items in this final, modified higher order model. Figure 1 illustrates the standardized loadings for factors, and squared multiple correlations indicating variance accounted for (*R*^2^) for each item.

**TABLE 2:** Items in the 35-item PMCQ.

Item	Old no.		Scale
1	1	I forget to do daily tasks such as paying bills, posting letters, or putting the garbage out	FB
2	2	I forget to pass important messages on to family, friends, or colleagues	FB
5	5	I put things in the wrong place, e.g., milk in the cupboard and sugar in the fridge	FB
6	6	In the middle of a sentence, I forget what I was going to say	FB
7	7	I forget important appointments	FB
8	9	When I am given a message to pass on, I forget what the message was	FB
10	11	I forget to do things that can be done in a sequence, e.g., buy a stamp, put the stamp on an envelope and post it	FB
13	16	When I have to do two things at once, I have trouble remembering to do both	FB
18	21	I remember to do things I need to do even if I am in the middle of another task*	FB
24	28	I do things twice because I forget that I have already done them, e.g., take a tablet twice	FB
25	29	I think that I have done things when I actually have not done them	FB
30	37	I forget to turn the stove or iron off	FB
19	22	I have trouble remembering directions or instructions	MC
20	23	I have trouble switching my attention between two different things, e.g., watching TV and talking to someone at the same time	MC
21	24	When I am tired, stressed, angry, or upset I forget to do things more often than normal	MC
22	25	I forget important dates, birthdays, or anniversaries	MC
27	31	I have trouble remembering the names of people and places	MC
28	32	I have trouble remembering recent events in my life	MC
31	38	I worry that my memory is getting worse	MC
32	39	I know that I am going to need a memory aid such as a note, list, or alarm	MC
33	40	It takes me longer to do mental tasks than it used to, e.g., crosswords	MC
34	41	I get frustrated with myself because I forget to do things that I was supposed to do	MC
35	42	I have trouble thinking of ways to help my memory	MC
3	3	There are times when I remember that I need to do something, but I cannot remember what it is	RF
4	4	I walk into a room and forget why I went there	RF
9	10	I forget to do things that I have started, e.g., hanging washing out once the washing machine has finished	RF
11	13	I forget where I have placed things, e.g., keys or money	RF
12	15	Seeing places or objects can remind me that I need to do something, but I cannot remember exactly what it is	RF
14	17	I forget to do things because I get carried away doing something else	RF
15	18	I find that I do not return to planned tasks if I get interrupted	RF
16	19	I forget to do some things that I have planned to do	RF
17	20	I forget things that I am supposed to be doing if I am anxious or worried about something	RF
23	27	I can only remember that I have a message to pass on when I see the person the message is for	RF
26	30	I tell people the same story because I forget that I have already told them	RF
29	33	I remember the main parts of instructions (e.g., buy milk) but I forget details (buy two liters of milk)	RF

FB, forgetting behaviors subscale; MC, memory concerns subscale; RF, retrieval failures subscale. *Reverse-scored items.

### Reliability

Cronbach’s alphas were calculated to evaluate the reliability of items in the final modified higher order PMCQ model. The 35 item PMCQ had excellent reliability (α = 0.95), while the subscales all had very good internal consistency (Forgetting behaviors: α = 0.89, Memory concerns: α = 0.88, Retrieval failures: α = 0.88). No further deletion of items would have improved the reliability of the PMCQ or its subscales.

### Normative Data for the PMCQ

Table 3 summarizes the mean scores, standard deviations, and standard error of the mean for the modified 35-item PMCQ and its subscales. As the PMCQ and its subscales each have different numbers of items, it is difficult to interpret and compare raw scores on these scales. Based on the procedures of Crawford et al. (2003) in the standardization of the PRMQ, T-scores were calculated so that the PMCQ and its subscales could be measured on a common metric. The t-distribution has a mean of 50 and standard deviation of 10. These T-scores conversion tables are provided in Supplementary Tables 2–5. Additional scoring information is also provided in the Supplementary Table 3 of the Supplementary Materials.

**TABLE 3:** Relationships between the PMCQ, its subscales, and performance-based PM tasks.

	*M*	*SD*	SEM	Range	1	2	3	4	5	6
1. PMCQ total	28.96	14.28	0.60	0–95	–					
2. Forgetting behaviors	7.33	4.60	0.19	0–33	0.86**	–				
3. Memory concerns	10.34	5.83	0.25	0–31	0.88**	0.60**	–			
4. Retrieval failures	11.29	5.43	0.23	0–32	0.92**	0.78**	0.68**	–		
5. EB naturalistic PM	0.35	0.48	–	0–1	−0.07	−0.14**	−0.01	−0.06	–	
6. TB naturalistic PM	0.73	0.94	–	0–2	−0.09	−0.12	−0.03	−0.10	0.04	–
7. Age	37.28	16.29		17–83	−0.21**	−0.31**	−0.12**	−0.36**	0.12**	0.26**

PMCQ, Prospective Memory Concerns Questionnaire; EB, event-based; TB, time-based. PMCQ total scores could range from 0 to 105, Forgetting Behaviors scores could range from 0 to 36, Memory Concerns scores could range from 0 to 33, Retrieval Failures could range from 0 to 36. **p < 0.001.

In terms of demographic differences on the PMCQ and its subscales, we found no significant differences between males and females (*ps* > 0.05) and therefore did not split normative data based on gender. We also did not split the normative data into age groups, as there was no theoretical rationale for splitting participants into arbitrary age groups. Instead, age was treated as a continuous variable, and therefore its relationships with the PMCQ and its subscales are reported as correlations in Table 3. As can be seen in the table, older age was associated with having fewer memory concerns on the PMCQ.

### Self-Report and Performance-Based PM

Scores on the modified 35-item self-report PMCQ, and performance-based event-based naturalistic PM task and time-based naturalistic PM task are summarized in Table 3. Scores on the PMCQ and its’ subscales indicated low levels of memory concerns in this sample of adults in the general population. For the event-based laboratory PM task, most participants forgot to carry out the task with only 34.9% of participants correctly writing their handedness in the comment box. Similarly, for the 219 participants who consented to the time-based naturalistic PM task, only 34.2% correctly remembered to text “hello” in the 12-h window the following day. An additional 4.6% remembered to send the text message at the correct time but did not include “hello” in their message.

To investigate relationships between the PMCQ, its subscales, age, the event-based naturalistic PM task, and the time-based naturalistic PM task, Spearman’s rho correlations were calculated (see Table 3). As can be seen in the table, the Forgetting Behaviors subscale that measures the frequency of PM failures had a weak significant relationship with performance on the event-based naturalistic PM task, but not the time-based naturalistic PM task. The total PMCQ scale, Memory Concerns subscale, and Retrieval failures subscale each had weak non-significant relationships with both performance-based PM tasks. The EB and TB performance-based tasks demonstrated divergent validity from one another, with weak and not significant correlations.

## Discussion

### Summary of Findings

The aim of this study was to examine the factor structure of the PMCQ for use with adults in the general population. We also investigated relationships between the PMCQ and performance-based measures of PM, specifically an event-based naturalistic PM task and time-based naturalistic PM task, to determine how the PMCQ can be used alongside these types of naturalistic tasks to comprehensively assess PM. We hypothesized that the PMCQ would have divergent validity with performance-based measures, with only weak relationships with the performance-based PM measures.

We found that the fit of the original three-factor structure of the 42-item PMCQ was poor. However, we modified the model by reducing the number of items to 35, relocating four items to alternate factors, and adding a higher order Prospective Memory factor to the three original factors. This modified PMCQ contains three subscales: The Forgetting Behaviors subscale contains 12 items measuring common PM failures. The Memory Concerns subscale includes 11 items that focus on general worries about PM and other cognitive processes involved in PM (e.g., failures of attention and RM). The Retrieval failures subscale is comprised of 12 items that measure problems with using retrieval cues effectively due to interruptions, absentmindedness, or not recognizing the cues. Thus, the PMCQ contains a unique constellation of items that fill some of the gaps in content relating to concerns about memory retrieval cues that were left by previous PM scales. The length of the PMCQ, at 35 items, makes the scales suitable for administration on its own, or as a part of a larger assessment battery. Moreover, the inclusion of the higher order factor allows the user to calculate an overall PMCQ score or separate scale scores relating to the three sub-factors.

This modified higher order PMCQ model had acceptable fit and reliability, and the provision of normative data makes it suitable for use in samples of adults in the general population. As predicted, relationships between the PMCQ and performance-based measures were weak. The only significant relationship found was between the PMCQ Forgetting Behaviors scale and the event-based naturalistic PM task. These findings provide some support for the divergent validity of the PMCQ and performance-based measures of PM and demonstrate the potential for the coadministration of these measures to comprehensively assess PM.

### Factor Structure of the PMCQ

The original three-factor model of the 42 item PMCQ had adequate fit in the original validation study (Sugden, 2015), but poor fit in this sample of healthy adults. It should be noted that in the original validation study, maximum likelihood estimation was used. However, in the current study, diagonal weighted least squares estimation was used, given more recent evidence that this method provides more robust estimates of model fit with ordinal data (Li, 2016). As such, we recommend that future studies test the models derived from the study using these more robust estimation methods to ensure that the higher order model derived in this study can be replicated.

Alternately, differences in the level of memory concerns across validation samples may have contributed to this finding. Samples in the original validation study and the current study both consisted of adults from the general population where it was assumed that participants would have low ratings of memory concerns. Consistent with these expectations, both samples had positively skewed distributions on the PMCQ and its subscales. However, the current sample had lower mean scores on the PMCQ and its subscales than the original validation sample, suggesting that they had less memory concerns.

Another possible explanation for differences between the original and modified structures is that the original sample completed a pencil and paper version of the PMCQ, whereas the current sample completed the PMCQ online. As Buchanan et al. (2005) found non-equivalent factor structures on pencil and paper versus online versions of the PMQ, it may be that the same effects have occurred with the PMCQ. Therefore, the original factor structure may be more suitable when pencil and paper versions of the PMCQ are used, although this will need to be investigated further. As the original model had poor fit in the current study, we modified the three-factor model, introducing a higher order PM factor and keeping the original factors, but with only 35 items. There were seven items that were deleted as they contributed only small amounts of variance to the model. It is likely that these items were contributing to the poor fit of the model as they focused on a general sense of being good at remembering or effectively using salient cues (e.g., item 26 “*I am good at remembering to do things on time*”), whereas remaining PMCQ items were framed as concerns about forgetting on more specific behaviors. Of the eliminated items, three were reverse scored, but it does not appear that there was a reverse scoring impact, as item 21 “*I remember to do things I need to do even if I am in the middle of another task*” was also reverse scored but contributed significant variance. An additional four items were moved to different factors based on modification indices and content analysis to ensure that changes were theoretically sound. Together, these changes resulted in a modified 35 item PMCQ scale that can reliably be used with adults in the general population.

There is some overlap in content between the PMCQ and other self-report measures of PM (i.e., the PMQ, PRMQ, CAPM, and BAPM), as common PM errors such as forgetting to pass on a message are assessed in all these questionnaires. There are also some similarities between the PMCQ and CAPM whereby both consider reasons for forgetting. What differentiates the PMCQ from these other scales though is its focus on concerns about PM and the inclusion of the Retrieval Failures scale which provides some insight into cognitive and metacognitive processes underlying retrieval of PM cues.

### Relationship With Performance-Based Measures

Relationships between the PMCQ, its subscales, and the event-based naturalistic PM task and time-based naturalistic PM task were weak, and the only significant relationship was between the Forgetting Behaviors subscale and event-based naturalistic PM task. Correlations between the PMQ, PRMQ, CAPM, and BAPM with various performance-based tasks have consistently been weak to moderate in size in previous studies (Sugden et al., 2021). Therefore, the weak relationships between PMCQ and performance-based measures in the current study were expected.

In this study, there did not appear to be any ceiling or floor effects for the performance-based tasks. However, as these tasks only included a single trial each, they would have been less able to discriminate between participants’ performance than performance measures that had more items. Performance on the event-based naturalistic and time-based naturalistic PM tasks was poor, with only approximately 34% of participants remembering to complete the tasks correctly. Yet, on the PMCQ, participants reported that they on average only “*sometimes*” had concerns about forgetting. These low levels of memory concerns would be expected in this sample of adults from the general population who would likely seldom forget the types of tasks captured in PMCQ items. To better discriminate between individuals with different levels of PM concerns and gain a full range of responses, recruitment of individuals with clinical impairments would be required.

The PMCQ was found to have adequate divergent validity with the time-based naturalistic and event-based PM performance measures to establish that they can assess different aspects of the multidimensional PM construct. As the PMCQ provides additional information to what was obtained in the event-based and time-based tasks, it can be used to complement these performance-based tasks to provide a more comprehensive assessment of PM. It is recommended that divergent validity is further established with other performance-based measures of PM, given that further evidence of the reliability and validity of these naturalistic PM tasks is needed. Although convergent validity of the PMCQ with the PRMQ was established in the development of the PMCQ (Sugden, 2015), it is recommended that the convergent validity of the modified 35-item PMCQ with self-reported measures of PM be investigated in future studies.

### Age Effects

The age PM paradox, whereby older adults perform poorly on laboratory tasks but outperform younger adults on naturalistic tasks that are more familiar to them, has frequently been reported (Henry et al., 2004; Ihle et al., 2012). In line with this paradox, in our study, older age was associated with higher levels of performance on both the event-based and time-based naturalistic tasks. Consistent with these findings, older age was also associated with fewer memory concerns on the PMCQ and its subscales.

On other self-report PM measures, similar age effects have been found on the CAPM, with older adults (aged 31–60 years) reporting fewer PM failures than younger adults (aged 15–30 years; Chau et al., 2007). Similarly, a trend toward older adults self-reporting fewer memory failures on the PRMQ was shown by (e.g., Rönnlund et al., 2008). However, the findings on age effects in self-reported PM are mixed, as other studies have failed to find significant correlations between age and PRMQ scores (Crawford et al., 2003) or differences between younger adults (aged 17–59) and older adults on the PRMQ (aged 59–93; Smith et al., 2000). Contributing to these mixed findings may be the use of different age brackets across studies. For this reason, we chose to investigate age as a continuous variable and recommend that future studies adopt a consistent framework for defining age groups to allow more direct comparison across studies.

Although the PMCQ did not correlate strongly with the naturalistic PM tasks, the finding of older adults having superior PM did hold across measurement methods. This is most likely due to the PMCQ items more closely resembling the naturalistic tasks that were carried out amongst participants daily activities rather than laboratory-based tasks (i.e., responding to target words on a computer screen) carried out in an artificial laboratory settings. Further supporting this finding of older adults having less self-reported and naturalistic PM errors is a diary study carried out by Niedźwieńska et al. (2020), who found that younger adults (aged 19–30) self-reported more everyday PM failures than both middle-aged adults (aged 35–55) and older adults (aged 61–80), who did not differ from one another.

Several explanations for older adults’ superior self-reported and naturalistic PM have been put forward. Crawford et al. (2003) noted that there is a tendency to base self-reports of memory on comparisons with peers. For older adults, this could include comparisons to peers experiencing neurocognitive decline due to dementia, resulting in older adults reporting fewer memory concerns than if they were comparing to younger adults. However, as older adults also performed better on the naturalistic PM tasks than younger adults, this suggests that even if age-peer comparisons have been made in older adults, that their self-reported PM does reflect their PM performance to some extent. An alternative explanation for older adults performing better on naturalistic PM tasks and reporting fewer concerns is that they make more effective use of retrieval cues and compensation strategies. Our findings lend some support to this argument, as older adults reported fewer issues with planning and cue retrieval on the PMCQ Retrieval failures subscale. Nevertheless, further research is required to these age effects and factors that may contribute to them.

### Limitations

The event-based and time-based naturalistic PM tasks each included a single trial and restricted scoring that reduced their ability to discriminate between good and poor performance. However, due to an inability to administer standardized performance measures such as the MIST (Raskin and Buckheit, 1998), CAMPROMPT (Wilson et al., 2005), or RPA-ProMem (Radford et al., 2011) face-to-face during COVID-19, the online event-based and time-based measures were used. It is recommended for future research that where possible, standardized performance-based measures that include multiple tasks be used to allow for greater generalization of findings.

Evidence for the reliability and divergent validity of the PMCQ has been presented in relation to the general population sample in this study. However, these findings cannot be generalized to clinical populations. Instead, studies investigating the factor structure, reliability, and validity of the PMCQ across various clinical populations is required. The collection of reference group data for clinical populations will allow for comparisons between reference groups and the general population normative data reported here to determine the degree of impairment in the reference groups.

In the event-based naturalistic PM task, although the instructions were highlighted in bold to draw the participants’ attention to the PM instructions, it is possible that participants may have still failed to notice these instructions. We recommend that future research using these types of tasks incorporates a measure to check that participants have read and understood the instructions to determine whether poor task performance is due to inattention to the instructions or poor PM performance.

Convenience sampling was used to recruit adults from the general population. It was assumed that these adults were healthy, however, without any neuropsychological screening, the cognitive status of participants cannot be confirmed. Despite this, attempts were made to stratify participants based on age and gender during participant recruitment, by actively targeting male participants and participants in age groups with fewer numbers to ensure that the sample was overall balanced in terms of age and gender to represent the population. To obtain a stratified sample, 95 male participants were recruited through paid participation websites, while another 84 participants were recruited through a student research participation pool. Although unlikely that these small financial incentives or course requirements would have impacted on PMCQ responses, there is a possibility that these participants may have been more motivated to succeed in the performance-based tasks than those recruited through other methods.

### Applications to Clinical Practice

As the PMCQ covers a broad range of dimensions, it has potential uses for examining factors underlying PM failures. The inclusion of normative data and standardized T-scores also permits easy interpretation of scores and facilitates comparison of performance across measures. The PMCQ fills a gap left by existing self-report measures of PM with its focus on concerns about memory and use of retrieval cues.

As divergent validity between the PMCQ and a time-based naturalistic PM task and event-based naturalistic PM task was established, it was shown that the PMCQ can be used alongside performance-based measures to provide a more comprehensive assessment of PM. The PMCQ might highlight the individual’s primary areas of concern about their memory or uncover underlying cognitive processes that may be involved in their PM failures. Using this information, individual variables may be controlled for or manipulated in performance-based tasks (e.g., altering the nature of the retrieval cue) to further elucidate reasons for forgetting. Information about primary areas of memory concern should also assist with treatment planning, as areas that have the greatest impact upon the individual’s life can be given priority when setting treatment goals (Roche et al., 2007). Finally, the PMCQ may aid in the evaluation of treatment outcomes, whereby memory concerns should be seen to reduce following treatments.

The PMCQ can be used in adults from the general population. However, further validation of the PMCQ is needed before it can be used in clinical populations to determine its sensitivity and specificity in identifying clinically relevant PM impairments. Without this validation, it is recommended that the PMCQ not be used for any diagnostic purposes, but instead, it should only be used to gather information about the individual’s memory concerns.

### Future Directions and Recommendations

To build on the validation of the PMCQ in this study, future research should aim to replicate the factor structure of the PMCQ obtained in this study and investigate the convergent validity of the PMCQ with other self-report measures of PM. In addition, validation of the PMCQ in clinical populations in future research would allow for the PMCQ to be used in clinical settings.

Informant-report versions of the PRMQ, CAPM, and BAPM have provided useful information about memory self-awareness issues, particularly in populations with cognitive impairments such as those with brain injury and dementia (Roche et al., 2002; Thompson et al., 2015). Given that the PMCQ focuses on an individual’s subjective concerns about their PM, an informant version of the PMCQ that could be used to corroborate some of these concerns would be worthwhile. In obtaining further information about the validity of the PMCQ, factors that may influence PM performance and self-reported PM should be investigated in more detail. This information will allow a deeper understanding of the construct that the PMCQ is measuring, so that it can be more effectively used alongside performance-based measures to comprehensively assess PM.

### Conclusion

This research produced the 35-item PMCQ, which can be use to investigate individuals’ forgetting behaviors, memory concerns, and failures to effectively use retrieval cues. The PMCQ, with its focus on memory concerns and retrieval processes, includes a unique set of items that differentiates it from existing self-report measures of PM. The PMCQ will be a useful tool for researchers and clinicians, as it can be used alongside performance-based measures of PM and other self-report measures of PM to provide a comprehensive assessment of PM in the adult general population.

## Data Availability Statement

The raw data supporting the conclusions of this article will be made available by the authors, without undue reservation.

## Ethics Statement

The studies involving human participants were reviewed and approved by the Charles Sturt University Human Research Ethics Committee. The patients/participants provided their written informed consent to participate in this study.

## Author Contributions

NS: conceptualization of the project, data analysis, writing of the manuscript, and critical review. MT and MK: conceptualization of the project, data analysis, and critical review. MW: data collection, writing of the manuscript, and critical review. All authors contributed to the article and approved the submitted version.

## Conflict of Interest

MT was employed by company Marathon Health. The remaining authors declare that the research was conducted in the absence of any commercial or financial relationships that could be construed as a potential conflict of interest.

## Publisher’s Note

All claims expressed in this article are solely those of the authors and do not necessarily represent those of their affiliated organizations, or those of the publisher, the editors and the reviewers. Any product that may be evaluated in this article, or claim that may be made by its manufacturer, is not guaranteed or endorsed by the publisher.
