# Supplementary material for: Validation of the Prospective Memory Concerns Questionnaire (PMCQ)
Source: Front Hum Neurosci. 2021 Aug 26;15:686850. doi: 10.3389/fnhum.2021.686850 (PMC8427764; doi:10.3389/fnhum.2021.686850)
Supplement: Supplementary file 1 [file Table_1.DOCX]

Table S1. *PMCQ Item Distributions*.

| Item |  | *M* | *SD* | *z* skew |
| --- | --- | --- | --- | --- |
| 1 | I forget to do daily tasks such as paying bills, posting letters, or putting the garbage out | 0.80 | 0.66 | 1.23 |
| 2 | I forget to pass important messages on to family, friends, or colleagues | 0.69 | 0.64 | 0.95 |
| 3 | There are times when I remember that I need to do something but I can’t remember what it is | 1.17 | 0.69 | 1.69 |
| 4 | I walk into a room and forget why I went there | 1.09 | 0.60 | 1.20 |
| 5 | I put things in the wrong place e.g., milk in the cupboard and sugar in the fridge | 0.39 | 0.60 | 0.43 |
| 6 | In the middle of a sentence I forget what I was going to say | 0.90 | 0.72 | 1.17 |
| 7 | I forget important appointments | 0.36 | 0.63 | 0.34 |
| 8 | If interrupted while doing something, I remember to finish it later* | 1.33 | 0.81 | 6.12 |
| 9 | When I am given a message to pass on, I forget what the message was | 0.55 | 0.65 | 0.65 |
| 10 | I forget to do things that I have started e.g., hanging washing out once the washing machine has finished | 0.77 | 0.73 | 1.01 |
| 11 | I forget to do things that can be done in a sequence e.g., buy a stamp, put the stamp on an envelope and post it | 0.27 | 0.50 | 0.27 |
| 12 | I am more likely to remember to do something if there is something to remind me e.g., an object or a person | 1.84 | 0.87 | -4.17 |
| 13 | I forget where I have placed things e.g., keys or money | 1.06 | 0.77 | 1.34 |
| 14 | Seeing places (e.g., a supermarket) or objects (e.g., a teapot) can remind me that I need to do something (e.g., buy sugar) | 1.52 | 0.80 | 4.73 |
| 15 | Seeing places or objects can remind me that I need to do something, but I can’t remember exactly what it is | 0.85 | 0.74 | 1.08 |
| 16 | When I have to do two things at once, I have trouble remembering to do both | 0.69 | 0.70 | 0.84 |
| 17 | I forget to do things because I get carried away doing something else | 1.18 | 0.73 | 1.68 |
| 18 | I find that I don’t return to planned tasks if I get interrupted | 0.77 | 0.70 | 1.05 |
| 19 | I forget to do some things that I have planned to do | 0.90 | 0.64 | 1.38 |
| 20 | I forget things that I am supposed to be doing if I am anxious or worried about something | 1.15 | 0.84 | 2.01 |
| 21 | I remember to do things I need to do even if I am in the middle of another task* | 1.47 | 0.70 | -6.89 |
| 22 | I have trouble remembering directions or instructions | 0.99 | 0.76 | 1.20 |
| 23 | I have trouble switching my attention between two different things e.g., watching TV and talking to someone at the same time | 1.03 | 0.91 | 1.46 |
| 24 | When I am tired, stressed, angry, or upset I forget to do things more often than normal | 1.35 | 0.82 | 4.13 |
| 25 | I forget important dates, birthdays, or anniversaries | 0.81 | 0.86 | 1.05 |
| 26 | I am good at remembering to do things on time* | 1.17 | 0.84 | 3.99 |
| 27 | I can only remember that I have a message to pass on when I see the person the message is for | 0.80 | 0.72 | 1.17 |
| 28 | I do things twice because I forget that I have already done them e.g., take a tablet twice | 0.35 | 0.58 | 0.35 |
| 29 | I think that I have done things when I actually have not done them | 0.52 | 0.66 | 0.52 |
| 30 | I tell people the same story because I forget that I have already told them | 0.87 | 0.81 | 0.98 |
| 31 | I have trouble remembering the names of people and places | 1.20 | 0.79 | 1.75 |
| 32 | I have trouble remembering recent events in my life | 0.58 | 0.72 | 0.65 |
| 33 | I remember the main parts of instructions (e.g., buy milk) but I forget details (buy two litres of milk) | 0.69 | 0.78 | 0.75 |
| 34 | I remember to do things by associating them with other things e.g., when I see John at work, I remember to pass on a message to him | 1.32 | 0.83 | 4.38 |
| 35 | I am more likely to remember to do things if I say them over and over to myself | 1.48 | 0.93 | 21.06 |
| 36 | I remember to do everyday tasks such as take medication, brush my teeth, or shower* | 0.50 | 0.81 | 0.50 |
| 37 | I forget to turn the stove or iron off | 0.33 | 0.61 | 0.30 |
| 38 | I worry that my memory is getting worse | 0.92 | 0.94 | 1.17 |
| 39 | I know that I am going to need a memory aid such as a note, list, or alarm | 1.35 | 0.93 | 2.96 |
| 40 | It takes me longer to do mental tasks than it used to e.g., crosswords | 0.66 | 0.82 | 0.69 |
| 41 | I get frustrated with myself because I forget to do things I was supposed to do | 0.87 | 0.88 | 1.07 |
| 42 | I have trouble thinking of ways to help my memory | 0.57 | 0.82 | 0.59 |

*Items are reverse scored.
