# Supplementary material for: Validation of the Prospective Memory Concerns Questionnaire (PMCQ)
Source: Front Hum Neurosci. 2021 Aug 26;15:686850. doi: 10.3389/fnhum.2021.686850 (PMC8427764; doi:10.3389/fnhum.2021.686850)
Supplement: Supplementary file 3 [file Table_3.docx]

**Prospective Memory Concerns Questionnaire (PMCQ)** **35-item scale**

**Instructions, scoring information, and items**

**Instructions for administration**

This questionnaire may be completed by the individual, or with the assistance of a researcher or clinician. Assistance should only include clarification of the meaning of items. Recall of the frequency of memory concerns should not be provided on behalf of the individual.

**Administration Instructions for the PMCQ**

The following questions include statements about your memory. Please read each item very carefully and decide how frequently you experience each of these behaviors. Please make sure that you answer each of the questions, even if they do not seem applicable to you.

**PMCQ subscales and scoring**

Note: item numbers refer to the modified 35 item PMCQ.

*PMCQ total scores*

To calculate the total PMCQ score, sum ratings of all 35 items to create a score out of 105. Alternately, an average score can be calculated by summing scores on all 35 items and dividing by 35. Item 21 is worded in the opposite direction to all other items and will therefore need to be reverse scored before calculating these scores.

*Forgetting Behaviors Subscale (FB)*

To calculate the Forgetting Behaviors subscale score, sum ratings on each of the 12 FB scores in the table below to create a score out of 36. Alternately, an average score can be calculated by summing scores on all 12 FB items and dividing by 12. Item 21 is worded in the opposite direction to all other items and will therefore need to be reverse scored before calculating these scores.

*Memory Concerns Subscale (MC)*

To calculate the Memory Concerns subscale score, sum ratings on each of the 11 MC scores in the table below to create a score out of 36. Alternately, an average score can be calculated by summing scores on all 11 MC items and dividing by 11.

*Retrieval Cue Subscale (RF)*

To calculate the Retrieval Failures subscale score, sum ratings on each of the 12 RF scores in the table below to create a score out of 36. Alternately, an average score can be calculated by summing scores on all 12 RF items and dividing by 12.

**T-scores**

Please refer to Tables S1-S5. Table S1 reports the mean, standard deviation, SEM, and range of PMCQ scores in the normative sample. Table S2 presents the raw score and the Z and T score equivalents for these raw scores on the PMCQ. Tables S3-S5 include the Z and T scores for the PMCQ subscales. T-scores have a mean of 50 and standard deviation of 10. Therefore, a T score of 60 indicates that the person scored one standard deviation above the mean of the normative sample. A T score of 30 suggest that the person scored 2 standard deviations below the average of the normative sample. These T-scores can be used to compare performance across the PMCQ and its subscales and other measures.

**Table S6. *PMCQ Items***

| Scale | Item |  |
| --- | --- | --- |
| FB | 1 | I forget to do daily tasks such as paying bills, posting letters, or putting the garbage out |
| FB | 2 | I forget to pass important messages on to family, friends, or colleagues |
| RF | 3 | There are times when I remember that I need to do something, but I cannot remember what it is |
| RF | 4 | I walk into a room and forget why I went there |
| FB | 5 | I put things in the wrong place e.g., milk in the cupboard and sugar in the fridge |
| FB | 6 | In the middle of a sentence, I forget what I was going to say |
| FB | 7 | I forget important appointments |
| FB | 8 | When I am given a message to pass on, I forget what the message was |
| RF | 9 | I forget to do things that I have started e.g., hanging washing out once the washing machine has finished |
| FB | 10 | I forget to do things that can be done in a sequence e.g., buy a stamp, put the stamp on an envelope and post it |
| RF | 11 | I forget where I have placed things e.g., keys or money |
| RF | 12 | Seeing places or objects can remind me that I need to do something, but I cannot remember exactly what it is |
| FB | 13 | When I have to do two things at once, I have trouble remembering to do both |
| RF | 14 | I forget to do things because I get carried away doing something else |
| RF | 15 | I find that I do not return to planned tasks if I get interrupted |
| RF | 16 | I forget to do some things that I have planned to do |
| RF | 17 | I forget things that I am supposed to be doing if I am anxious or worried about something |
| FB | 18* | I remember to do things I need to do even if I am in the middle of another task* |
| MC | 19 | I have trouble remembering directions or instructions |
| MC | 20 | I have trouble switching my attention between two different things e.g., watching TV and talking to someone at the same time |
| MC | 21 | When I am tired, stressed, angry, or upset I forget to do things more often than normal |
| MC | 22 | I forget important dates, birthdays, or anniversaries |
| RF | 23 | I can only remember that I have a message to pass on when I see the person the message is for |
| FB | 24 | I do things twice because I forget that I have already done them e.g., take a tablet twice |
| FB | 25 | I think that I have done things when I actually have not done them |
| RF | 26 | I tell people the same story because I forget that I have already told them |
| MC | 27 | I have trouble remembering the names of people and places |
| MC | 28 | I have trouble remembering recent events in my life |
| RF | 29 | I remember the main parts of instructions (e.g., buy milk) but I forget details (buy two litres of milk) |
| FB | 30 | I forget to turn the stove or iron off |
| MC | 31 | I worry that my memory is getting worse |
| MC | 32 | I know that I am going to need a memory aid such as a note, list, or alarm |
| MC | 33 | It takes me longer to do mental tasks than it used to e.g., crosswords |
| MC | 34 | I get frustrated with myself because I forget to do things that I was supposed to do |
| MC | 35 | I have trouble thinking of ways to help my memory |

*Items are reverse scored.
